# COVID-19 Increased Residency Applications and How Virtual Interviews Impacted Applicants

**DOI:** 10.7759/cureus.26096

**Published:** 2022-06-19

**Authors:** Alex M Meyer, Alexander A Hart, Jerrod N Keith

**Affiliations:** 1 Carver College of Medicine, University of Iowa, Iowa City, USA; 2 Plastic and Reconstructive Surgery, University of Iowa Hospitals and Clinics, Iowa City, USA

**Keywords:** finance, virtual interviews, applications, residency selection, covid-19

## Abstract

Background

The number of residency applications submitted by medical students has risen at an alarming rate, causing increased cost of applications and subsequent interview travel. These both contribute to increased cost for medical students. In light of these concerns, specialty governing bodies have proposed ideas to fight these trends including, application limits, interview limits, using a preference signaling system, and continuing virtual interviews. During the Covid-19 pandemic, all residency interviews were performed virtually, essentially making travel expenses negligible. However, this created a new concern with regards to assessing program and applicant compatibility, as compared to in-person interactions and did nothing to combat the increases in application numbers. Therefore, we want to critically assess the effects of virtual interviews on number of applications submitted, number of interview invites received, and number of interviews attended. We also aim to analyze how applicants viewed the virtual process.

Methods

600 medical students were eligible to participate. 456 students from years 2018-2020 were eligible to be surveyed following the NRMP match. 144 students were eligible to be surveyed following 2021 NRMP match. The survey was distributed to medical school graduates just prior to graduation and asked how many programs each student applied to, how many interview invites they received, and how many interviews they attended. The 2021 survey also asked, “How did virtual interviews affect your interview experience?” The quantitative results were compared with student's t-test and qualitative results are presented below.

Results

The average number of programs each applicant applied to increased from 35.4 to 47.7 (p-value=0.002) when residency interviews switched from in-person to virtual. However, interview invites received and interviews attended did not change (16.8 vs 16.3, p-value=0.91, 11.8 vs 12.7, p-value=0.18). There were 188 participants in the in-person interview group (response rate=41.2%) and 128 participants in the virtual interview group (response rate=83.3%). The standard deviation and range also increased for number of applications, number of interview invites received, and number of interviews attended.

There were 123 responses to the free response question. 36 had a positive experience, 44 were neutral, 47 were negative. The positive themes included 15 noted less expenses, 18 noted more convenient/less time, and 18 were able to attend more interviews. Negative themes included, 38 noted difficulty assessing program fit, 19 wanted to see the program or city in person, eight had increased interest in home/local programs, six found it difficult to make connections or stand out.

Conclusion

Sixty-three percent of students reported a positive or neutral experience with virtual interviews. Students applied to more programs when interviews were virtual, but did not receive more interview invites or attend more interviews. These results suggest that virtual interviews are sufficient to conduct residency interviews, however the number of applications continues to rise with no increase in the number interview invites received or number of interviews attended. The increase in the standard deviation and range for all three variables may point to some applicants being able to get more invites and attend more interviews leaving less available spots for other applicants.

## Introduction

Medical students are currently applying to more residency programs than they ever have before, and we are continuing to see an increase each year [1,2]. Increased application numbers can be fueled by a fear of not matching and can lead to increased costs for students [1,3]. During the 2020-2021 Electronic Residency Application Service (ERAS) cycle, due to the Covid-19 pandemic, the Association of American Medical Colleges (AAMC) recommended doing all interviews virtually [4].

Subsequently, changes have been proposed for upcoming interview cycles, including continuing with virtual interviews, and implementing application/interview limits, in an attempt to maximize efficiency and minimize cost [1,5,6]. However, one concern for students is they may have adequate exposure to programs, allowing them to confidently make an informed decision. Applicants’ thoughts on virtual interviews have been an emerging research topic in an attempt to obtain guidance on the ideal process for future interview cycles [7]. 

While the trend of increased application numbers for individual specialties has been published [8-16], the comparison between applications, interview invites, and interviews attended across the spectrum of virtual and in-person interviews has not been examined.

This study aims to identify variations between the number of residency applications completed, interview invites received, and interviews attended during the two separate styles of interviews: virtual and in-person. We also aim to summarize applicants’ thoughts and feelings toward virtual interviews, identifying themes that may lead to future improvements.

## Materials and methods

Inclusion and exclusion criteria

All graduating medical students from the years 2018 to 2021 were eligible to participate (601 students). For the in-person interview group, 457 students from the years 2018 to 2020 were invited to participate in this study following the National Resident Matching Program (NRMP) match. For the virtual interview group, 144 students were invited to participate in this study following the 2021 NRMP match. No participants were excluded from the study other than those that chose not to complete the survey. This study was reviewed by the University of Iowa Institutional Review Board at a single United States allopathic medical school and granted IRB exemption due to the survey nature of the study (IRB number: 201308718). Informed consent was waived by the IRB, however, it was readily available for all participants.

Study design

This is a retrospective cohort study with the experimental groups being the in-person interview group and virtual interview group. We retrospectively reviewed the data following the match process and participants were separated into the two experimental groups according to their graduation year and the type of interviews that they underwent i.e., in-person interviews for the batch of 2018 to 2020 or virtual interviews for the batch of 2021.

Data collection

Paper surveys were distributed to participants when they picked up their graduation materials by members of the research team. They were completed and returned immediately. All surveys asked, the number of programs each student applied to, interview invites received, and interviews attended. The 2021 survey also asked, “How did virtual interviews affect your interview experience?” The survey responses were reviewed by the research team and deidentified in an excel database. The quantitative data was recorded as numerical values. The qualitative data was recorded exactly as the participant had written it for further analysis. The qualitative free response answers were categorized into positive, negative, or neutral responses as well as seven emerging themes. Each response could have more than one comment classified to each theme. If a student listed a positive and negative response, then it was classified as neutral. Each response was classified as a single data point as positive, negative, or neutral. Then all other comments were treated individually totaling 124 individual data points. Any responses that simply stated, “virtual interviews were not good,” “virtual interviews had no effect,” or “virtual interviews were good,” were only classified as negative, neutral, or positive accordingly, and not included in themes analysis. The themes that were considered positive were fewer expenses, more convenience/less time, and the ability to attend more interviews. Negative themes included difficulty assessing program fit, wanting to see more of the city/hospital, increased interest in home/local programs, and difficulty making connections/standing out. Assigning comments to positive, negative, or neutral and developing themes was performed independently by two authors (AM, AH). Any discrepancies were discussed, and an agreement was obtained.

Statistical analysis

We compared the number of programs applied to, interview invites received, and interviews attended for the in-person interview cohort to the virtual interview cohort using Student’s t-test. Statistical analysis was performed using Stata 19 (College Station, TX). Qualitative data was only available for the virtual interview cohort, therefore it is discussed and displayed visually in the results section. 

## Results

Response rates for years 2018 to 2020 were (188/457) 41.1%, and 83.3% (124/144) for 2021. There were no differences in age, gender, or ethnicity between the two experimental groups (Table 1). There was an increase in the number of programs applied to in the virtual interview group. This group applied to an average of 47.7 programs per applicant compared to 35.4 programs per applicant for the in-person interview group. There was no difference in the number of interview invites received between the virtual interview group and the in-person interview group (16.3 vs 16.8 p-value=0.91) (Table 2). There was also no difference in the average number of interviews attended in the virtual interview group compared to the in-person interview group (12.7 vs 11.8 p-value=0.18) (Table 2).

**Table 1:** Demographic information for each study group

	2018 to 2020 (n=188)	2021 (n=124)
Age Average ± standard deviation	27.6 ± 2.3	27.3 ± 1.8
Men (%)	88 (46.8%)	60 (47.2%)
Race (%) Caucasian	141 (75%)	98 (77.1%)
African American	5 (2.7%)	3 (2.4%)
Asian	33 (17.6%)	20 (15.7%)
Hispanic/Latino	9 (4.8%)	6 (4.7%)

**Table 2:** Number of programs applied to, interview invites received, and interviews attended for in-person interviews compared to virtual interviews

	In-person interviews (n=188)	Virtual interviews (n=127)	p-values
Number of programs applied	35.4	47.7	0.002
Standard deviation of the number of programs applied	19.3	32.6	
Minimum-maximum number of programs applied minimum-maximum	3-120	4-235	
Interview invites received	15.9	16.5	0.91
Standard deviation of the interview invites received	7.6	10.7	
Minimum-maximum interview invites received	3-57	2-60	
Interviews attended	11.8	12.7	0.18
Standard deviation of the interviews attended	4.2	5.5	
Minimum-maximum interviews attended	1-25	1-30	

**Figure 1:**
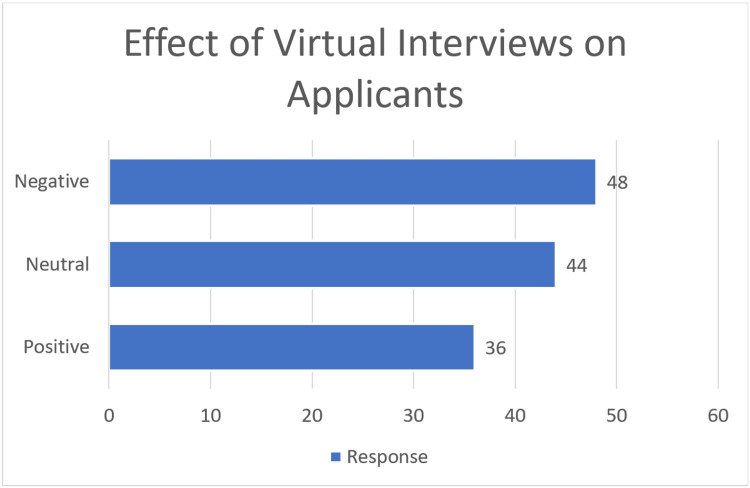
Break down of the positive, neutral, or negative comments on virtual interviews from the 128 responses

**Figure 2:**
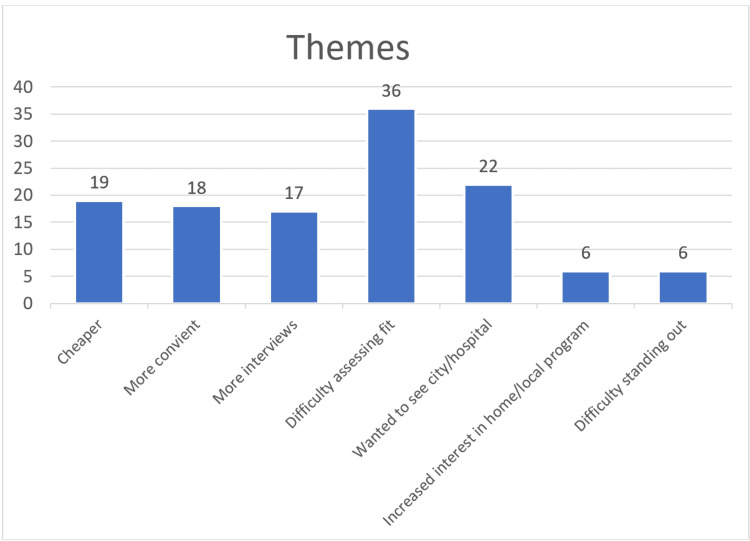
Most common themes from the 124 free text responses

There were 128 responses to the free-response question (response rate=128/144, 88.9%). Thirty-six had a positive experience, 44 were neutral, and 48 were negative (Figure 1). Therefore, 62.5% of respondents noted a positive or neutral experience with virtual interviews. Of the 128 overall responses, 124 comments were categorized into either one of the three positive themes or one of the four negative themes. The positive themes included 19 noting fewer expenses/cheaper, 18 noted more convenient/less time, and 17 were able to attend more interviews (Figure 2). Negative themes included 36 indicating difficulty assessing program fit, 22 wanted to see the program or city in person, six had increased interest in home/local programs, and six said it was difficult to make connections or stand out (Figure 2).

## Discussion

We saw an increase in the number of applications submitted during virtual interviews, but we did not see an increase in interview invites received or interviews attended. As mentioned above, this was likely because interview invites are a relatively fixed variable. We postulate that applicants likely were aware of the upcoming decreased cost of virtual interviews and may have already set aside or borrowed funds to complete the interview cycle, therefore allowing more spending on the total number of submitted applications. Even with a slight increase in application fees, the process was considerably cheaper than in prior cycles. This further supports the recommendations for application limits, as the increase in applications had diminishing returns, as it did not result in increased interview invites or interviews attended [1,5,6]. As mentioned above, it can be disadvantageous to many students.

Overall, almost two-thirds of participants reported a positive or neutral experience with virtual interviews. Therefore, in conjunction with the decreased time and cost, and consistent with other published findings [7], virtual interviews may be a reasonable option for future interview cycles. The most prevalent positive responses were fewer expenses, more convenient/less time commitment, and the ability to attend more interviews.

The decreased expenses are expected and primarily due to the lack of travel expenses. Fogel et al. found that the expenses for each typical orthopedic surgery residency interview were $250 to $499. Around 13% of those study participants spent more than $7,500 on interviews, and 41% stated they declined interviews due to financial reasons [17]. Additionally, Blackshaw et al. found that each in-person emergency medicine residency interview costs an average of $342 for an average total of $8,312 on applications and interviews [18]. Orthopedic surgery, neurosurgery, emergency medicine, ophthalmology, internal medicine, radiology, and otolaryngology have all published papers highlighting the increased cost of applying to residency in each of their particular specialties [8,10-12,16,18,19]. Continuing virtual interviews certainly will decrease the financial burden placed on students.

Along with the cost savings, many respondents reported increased convenience and decreased time commitment with virtual interviews. These findings are associated with students reporting the ability to attend more interviews. This was classified as a positive in this study since more interviews will likely increase the likelihood of an applicant matching a residency position. However, as demonstrated by Whipple et al., while applying for more programs can be advantageous for one applicant, it can be a disadvantage to the group of applicants, overall. When the entire cohort applies to more programs the benefit of increased matching likelihood is lost, yet the overall cost is increased [20]. They found when all applicants applied to the maximal number of programs it led to a poorer result for the majority of students. These findings make sense because if one applicant applies to more programs than the other applicants, they are more likely to receive more interviews. If they receive more interviews, they can have a longer rank list thus increasing their chance of matching to one of those programs. However, the total number of interview slots available is relatively fixed. If stronger applicants complete more interviews, they may cause the undesired effects of decreasing the number of interviews available for average or below-average applicants, therefore decreasing their likelihood of matching into their desired specialty. For example, if a strong applicant is now able to attend 20 instead of 15 interviews, another applicant who previously may have received and attended 10 interviews may now only receive five interviews. In both theoretical scenarios, the average is unchanged at 12.5 interviews, but the applicant with more interviews and a longer rank list is more likely to match. As seen in our study, the average number of interviews attended for in-person and virtual interviews was similar. This was in the context of multiple students reporting being able to attend more interviews, yet the average number of interviews remained the same. This phenomenon of some students getting more interviews while some receiving fewer occurred in our study population, as seen with the much larger standard deviation and range during virtual interviews (Table 2). These findings are the basis of capping residency interviews at 12 per applicant, as proposed by Morgan et al. [5]. We find this to be one of the most concerning issues with virtual interviews as they currently are being conducted. Average but competitive applicants may not match within the current algorithm just because they were unable to receive the same number of interviews as they previously might have during in-person interviews.

The negative aspects of virtual interviews demonstrated in this study are difficulty assessing program fit, wanting to see the program or city in person, increased interest in home/local programs, and difficulty in making connections or standing out. Assessing fit may be the more difficult item to rectify using virtual platforms due to the lack of organic in-person interactions. One potential solution to this issue is one round of virtual interviews, followed by another round of in-person interviews [21]. The opportunity to see more of the city or hospital facilities is an easier obstacle to overcome. With additional time and careful planning, programs may be able to provide more adequate videos and photos of the hospital facilities and surrounding city. Some programs such as the Rutgers New Jersey Medical School Med-Peds residency program are using virtual reality with 66% of respondents stating that virtual reality was superior or non-inferior to in-person tours [22]. We classified increased interest in home/local programs as a negative response because it likely decreases diversity in residency programs. A lack of diversity in medical training has been highlighted in both orthopedic and otolaryngology literature [23,24]. Therefore, in an attempt to continue to decrease these disparities, we classified it as a negative response in our survey. These obstacles can be addressed by interviewing more applicants from other regions and other medical schools. However, before virtual interviews, as shown by Loh et al., students were already more likely to match in the same region they attended medical school [25]. The final negative response students reported was difficulty making connections or standing out. However, as students may feel this is a concern, each student is given more equal opportunity for total virtual face time, as compared to informal dinners and gatherings. Additionally, some students may have similar feelings about in-person interviews. There is also published literature by Sarac et al. on how to optimize the virtual interview experience and how to best prepare [26]. Therefore, we feel like this is a real, but correctable concern with virtual interviews.

A potential limitation to this study is the classification of positive, negative, or neutral responses. For one student, increased interest in home/local programs may be positive, while for another it may be negative. We also classified responses with a positive and negative theme as neutral responses. For example, decreased cost and difficulty assessing fit were both positive and negative responses, respectively, which were analyzed as such and were also included in the neutral group. For each student, these factors hold different weight, and it is impossible to accurately discern how much each factor plays into a student’s decision. In the future, we want to examine the trends over time with a larger multi-institutional study population.

## Conclusions

In conclusion, conducting virtual interviews correlated with an increased number of residency program applications, but not an increased number of interview invites nor interviews attended. Overall, most applicants felt the virtual interviews did not cause a negative interview experience and saved considerable expenses. Therefore, we conclude that with concentrated efforts to improve concerns identified in this study such as assessing fit and being able to see more of the hospital/city even virtually, virtual interviews are an effective method for conducting residency interviews. 

## References

[1] Weissbart SJ, Kim SJ, Feinn RS, Stock JA (2015). Relationship between the number of residency applications and the yearly match rate: time to start thinking about an application limit?. J Grad Med Educ.

[2] (2020). ERAS Statistics | AAMC. https://www.aamc.org/media/39306/download.

[3] (2020). The cost of applying for medical residency | AAMC. http://residents.aamc.org/financial-aid/article/cost-applying-medical-residency/.

[4] (2021). Medical education | AAMC. https://www.aamc.org/what-we-do/mission-areas/medical-education.

[5] Morgan HK, Winkel AF, Standiford T (2021). The case for capping residency interviews. J Surg Educ.

[6] Gabrielson AT, Kohn JR, Sparks HT, Clifton MM, Kohn TP (2020). Proposed changes to the 2021 residency application process in the wake of COVID-19. Acad Med.

[7] Seifi A, Mirahmadizadeh A, Eslami V (2020). Perception of medical students and residents about virtual interviews for residency applications in the United States. PLoS One.

[8] Venincasa MJ, Cai LZ, Gedde SJ, Uhler T, Sridhar J (2020). Current applicant perceptions of the ophthalmology residency match. JAMA Ophthalmol.

[9] Trikha R, Keswani A, Ishmael CR, Greig D, Kelley BV, Bernthal NM (2020). Current trends in orthopaedic surgery residency applications and match rates. J Bone Joint Surg Am.

[10] Sweet ML, Williams CM, Stewart E, Chudgar SM, Angus SV, Kisielewski M, Willett LL (2019). Internal medicine residency program responses to the increase of residency applications: differences by program type and characteristics. J Grad Med Educ.

[11] Rozenshtein A, Gilet AG, Griffith B, Kamran A, Wiggins EF 3rd, Anderson JC (2018). Radiology residency match: the cost of being in the dark. Acad Radiol.

[12] Li NY, Gruppuso PA, Kalagara S, Eltorai AE, DePasse JM, Daniels AH (2019). Critical assessment of the contemporary orthopaedic surgery residency application process. J Bone Joint Surg Am.

[13] Huang RD, Lutfy-Clayton L, Franzen D (2020). More is more: drivers of the increase in emergency medicine residency applications. West J Emerg Med.

[14] Huang MM, Clifton MM (2020). Evaluating urology residency applications: what matters most and what comes next?. Curr Urol Rep.

[15] Finkler ES, Fogel HA, Kroin E, Kliethermes S, Wu K, Nystrom LM, Schiff AP (2016). Factors influencing the number of applications submitted per applicant to orthopedic residency programs. Med Educ Online.

[16] Chang CW, Erhardt BF (2015). Rising residency applications: how high will it go?. Otolaryngol Head Neck Surg.

[17] Fogel HA, Liskutin TE, Wu K, Nystrom L, Martin B, Schiff A (2018). The economic burden of residency interviews on applicants. Iowa Orthop J.

[18] Blackshaw AM, Watson SC, Bush JS (2017). The cost and burden of the residency match in emergency medicine. West J Emerg Med.

[19] Agarwal N, Choi PA, Okonkwo DO, Barrow DL, Friedlander RM (2017). Financial burden associated with the residency match in neurological surgery. J Neurosurg.

[20] Whipple ME, Law AB, Bly RA (2019). A computer simulation model to analyze the application process for competitive residency programs. J Grad Med Educ.

[21] Zaki MM, Nahed BV (2020). Utilizing virtual interviews in residency selection beyond COVID-19. Acad Med.

[22] Zertuche JP, Connors J, Scheinman A, Kothari N, Wong K (2020). Using virtual reality as a replacement for hospital tours during residency interviews. Med Educ Online.

[23] Ramirez RN, Franklin CC (2019). Racial diversity in orthopedic surgery. Orthop Clin North Am.

[24] Newsome H, Faucett EA, Chelius T, Flanary V (2018). Diversity in otolaryngology residency programs: a survey of otolaryngology program directors. Otolaryngol Head Neck Surg.

[25] Loh AR, Joseph D, Keenan JD, Lietman TM, Naseri A (2013). Predictors of matching in an ophthalmology residency program. Ophthalmology.

[26] Sarac BA, Calamari K, Janis J (2020). Virtual residency interviews: optimization for applicants. Cureus.

